# Supplementary material for: Clusters of polymorphic transmembrane genes control resistance to schistosomes in snail vectors
Source: eLife. 2020 Aug 26;9:e59395. doi: 10.7554/eLife.59395 (PMC7494358; doi:10.7554/eLife.59395)
Supplement: Supplementary file 1. [file elife-59395-supp1.docx]

**Supplementary File 1.** Supplementary Tables.

*Tennessen et al. 2020, “Clusters of polymorphic transmembrane genes control resistance to schistosomes in snail vectors”*

*Supplementary file 1A. Peaks in genome-wide association study results (Figure 1A).*

| **Linkage group** | **Candidate region** | **Highest per-window F_ST_** | **Contig(s) in *homR* assembly overlapping candidate region** | **Contigs containing windows with F_ST_ within 25% of highest per-window F_ST_ value for that linkage group** |
| --- | --- | --- | --- | --- |
| VII | RADres | 0.055 | R-19 | R-19, R-22, R-301, R-318, R-392, R-1202, R-1212 |
| IX | sod1 | 0.051 | R-20 | R-0, R-20, R-50, R-89, R-344, R-427, R-485, R-486 |
| XII | PTC2 | 0.247 | R-35, R-304 | R-35, R-304 |

*Supplementary file 1B. Hypothetical map distance between BS-90 RAPD marker and perfectly penetrant causal locus. While PTC2 is not perfectly penetrant in 13-16-R1, it could be in a cross between snail lines (Knight et al. 1999). Distances between 6 and 33 cM are plausible for the 54 resistant F_2_ progeny of which 90% carry a dominant marker (Knight et al. 1999).*

| **N = number of resistant progeny (expected genotypes at dominant causal locus)** | **Map distance between marker and causal locus (cM)** | **X̄ = expected number of recombinants (heterozygotes + homozygotes)** | **X̄/N** | **P-value (binomial probability given X̄/N)** |
| --- | --- | --- | --- | --- |
| 54 (36 heterozygous, 18 homozygous) | 6 | 2.10 (2.03 + 0.06) | 0.039 | 0.06 |
| 54 (36 heterozygous, 18 homozygous) | 17 | 5.60 (5.08 + 0.52) | 0.104 | 0.5 |
| 54 (36 heterozygous, 18 homozygous) | 33 | 9.92 (7.96 + 1.96) | 0.184 | 0.05 |

*Supplementary file 1C. PacBio genome assemblies.*

| **Genome** | **N50** | **Coverage** | **Total length** | **Contigs** | **Longest contig (size)** | **PTC2 (position on each contig in kb)** |
| --- | --- | --- | --- | --- | --- | --- |
| *homR* | 743 kb | 77.9× | 810.9 Mb | 2718 | R-0 (3.6 Mb) | R-35 (1595-1938), R-304 (635-765) |
| *homS1* | 2598 kb | 58.4× | 852.0 Mb | 927 | S1-0 (10.0 Mb) | S1-15 (1611-1895) |
| *homS2* | 395 kb | 46.0× | 767.7 Mb | 3492 | S2-0 (2.1 Mb) | S2-78 (0-202), S2-773 (243-324) |

*Supplementary file 1D. Genes in PTC2.*

| **Gene** | **Reference genome *BglaB1* closest match** | **TM1** | **Length (codons)** | **Haplotypes present on** |
| --- | --- | --- | --- | --- |
| 1 | *BGLB030379* | yes | 335-351 | R, S1, S2 |
| 2 | *BGLB038659* | yes | 376-530 | R, S1, S2 |
| 3 | *BGLB038659* | no | 187 | S1, S2 |
| 4 | *BGLB027019* | yes | 337 | R, S1, S2 |
| 5 | *BGLB031455* | yes | 298-304 | R, S1, S2 |
| 6 | *BGLB031125* | no | 127 | S2 |
| 7 | *BGLB029693* | yes | 286 | S1 |
| 8 | *BGLB029318* | yes | 218 | S2 |
| 9 | *BGLB018951* | yes | 316-346 | R, S1, S2 |
| 10 | *BGLB016855* | yes | 166 | R |
| 11 | *BGLB029318* | no | 224 | R |

*Supplementary file 1E. Expression of PTC2 genes. As we only have a single estimate per haplotype for each gene, we cannot quantify expression in a rigorous way. However, these point estimates suggest that there is little difference in expression among alleles, but moderate differences among loci. Expression on each haplotype, where applicable, is given in Reads Per Kilobase of transcript, per Million mapped reads (RPKM).*

| **Gene** | **R** | **S1** | **S2** |
| --- | --- | --- | --- |
| 1 | 0.70 | 1.01 | 0.64 |
| 2 | 0.15 | 0.56 | 0.10 |
| 3 | NA | 0.03 | 0.00 |
| 4 | 1.22 | 1.17 | 2.46 |
| 5 | 0.45 | 0.08 | 0.26 |
| 6 | NA | NA | 0.54 |
| 7 | NA | 0.34 | NA |
| 8 | NA | NA | 0.04 |
| 9 | 8.62 | 23.53 | 5.90 |
| 10 | 0.31 | NA | NA |
| 11 | 0.05 | NA | NA |

*Supplementary file 1F. Primers used to genotype PTC2.*

| **Primer Name** | **Orientation** | **PCR product distinguishing aspect** | **Sequence** | **Tm °C** | **Genotypes** |
| --- | --- | --- | --- | --- | --- |
| PB35_1696k_F | forward | Length (R: 497bp, S1: 185bp) | GGTTCTCGCTTTTTATTGGCTTTTG | 60 ºC | R, S1 |
| PB35_1696k_R | reverse | Length (R: 497bp, S1: 185bp) | TTAGACGCACCCAAGGATCTC | 60 ºC | R, S1 |
| VB13_859k_Fb | forward | Presence/absence (S2: 267bp) | ACAAATGGGGCAGTTACACTGTTTAC | 58 ºC | S2 |
| VB13_859k_Rb | reverse | Presence/absence (S2: 267bp) | AGCGAAATGTGAGATTGGTTATGTTG | 58 ºC | S2 |
| VB13_868k_Fb | forward | Variants from Sanger sequence | TCTTTTCACTAAAGCCGCACAAGTT | 58 ºC | R, S1, S2 |
| VB13_868k_Rb | reverse | Variants from Sanger sequence | CCTACGTTCTCAATATCAACGGGAA | 58 ºC | R, S1, S2 |
