# Supplementary material for: Clusters of polymorphic transmembrane genes control resistance to schistosomes in snail vectors
Source: eLife. 2020 Aug 26;9:e59395. doi: 10.7554/eLife.59395 (PMC7494358; doi:10.7554/eLife.59395)
Supplement: Transparent reporting form [file elife-59395-transrepform.pdf]

eLIFE
1st Floor
24 Hills Road
Cambridge CB2 1JP, UK
P 01223 855340
W elifesciences.org
T @elife

eLife's transparent reporting form

We encourage authors to provide detailed information *within their submission* to facilitate the interpretation and replication of experiments. Authors can upload supporting documentation to indicate the use of appropriate reporting guidelines for health-related research (see EQUATOR Network), life science research (see the BioSharing Information Resource), or the ARRIVE guidelines for reporting work involving animal research. Where applicable, authors should refer to any relevant reporting standards documents in this form.

If you have any questions, please consult our Journal Policies and/or contact us: editorial@elifesciences.org.

Sample-size estimation

- You should state whether an appropriate sample size was computed when the study was being designed
- You should state the statistical method of sample size computation and any required assumptions
- If no explicit power analysis was used, you should describe how you decided what sample (replicate) size (number) to use

Please outline where this information can be found within the submission (e.g., sections or figure legends), or explain why this information doesn't apply to your submission:

For the samples used in pooled sequencing (1200 snails), the sample size was chosen based on simulated data, as explained in Materials and Methods, "Genome-wide scan of 13-16-R1" section. To generate these 600 infected and 600 uninfected snails, we challenged 1700 snails, and we subsequently genotyped the majority (1570) of these at our candidate locus (not all samples could be genotyped for logistical reasons, e.g. insufficient high-quality DNA remaining). These 1570 are expected to provide even greater statistical power than the 1200 samples in pooled sequencing, both because of the large number and because full diploid genotypes provide more information. If these results had been ambiguous, we would have challenged and genotyped more snails as needed. The validation set of 392 snails was a sample of convenience in that the samples had previously been phenotyped, but as we demonstrate this sample was enough to confirm the effect with statistical significance (see Results and Discussion).

Replicates

- You should report how often each experiment was performed
- You should include a definition of biological versus technical replication
- The data obtained should be provided and sufficient information should be provided to indicate the number of independent biological and/or technical replicates
- If you encountered any outliers, you should describe how these were handled
- Criteria for exclusion/inclusion of data should be clearly stated
- High-throughput sequence data should be uploaded before submission, with a private link for reviewers provided (these are available from both GEO and ArrayExpress)

eLife Sciences Publications, Ltd is a limited liability non-profit non-stock corporation incorporated in the State of Delaware, USA, with company number 5030732, and is registered in the UK with company number FC030576 and branch number BR015634 at the address 1st Floor, 24 Hills Road, Cambridge CB2 1JP | August 2014

Please outline where this information can be found within the submission (e.g., sections or figure legends), or explain why this information doesn't apply to your submission:

The pooled whole-genome sequencing and subsequent genotyping of the same samples constitutes a single biological replicate. The second confirmatory replicate was the independent set of previously-phenotyped samples. There were individual outlier variants showing high Fst between the pools which could represent false positives, so to minimize this kind of noise we analyzed the results by genomic window rather than by individual variant. This is all explained in Materials and Methods, "Genome-wide scan of 13-16-R1" section. All high-throughput data are available in NCBI, with BioProject numbers indicated in Materials and Methods.

Statistical reporting

- Statistical analysis methods should be described and justified
- Raw data should be presented in figures whenever informative to do so (typically when N per group is less than 10)
- For each experiment, you should identify the statistical tests used, exact values of N, definitions of center, methods of multiple test correction, and dispersion and precision measures (e.g., mean, median, SD, SEM, confidence intervals; and, for the major substantive results, a measure of effect size (e.g., Pearson's r, Cohen's d)
- Report exact p-values wherever possible alongside the summary statistics and 95% confidence intervals. These should be reported for all key questions and not only when the p-value is less than 0.05.

Please outline where this information can be found within the submission (e.g., sections or figure legends), or explain why this information doesn't apply to your submission:

Statistics and p-values are reported throughout the Results and Discussion where appropriate. Details of statistical tests are explained in Materials and Methods.

(For large datasets, or papers with a very large number of statistical tests, you may upload a single table file with tests, Ns, etc., with reference to sections in the manuscript.)

Group allocation

- Indicate how samples were allocated into experimental groups (in the case of clinical studies, please specify allocation to treatment method); if randomization was used, please also state if restricted randomization was applied
- Indicate if masking was used during group allocation, data collection and/or data analysis

Please outline where this information can be found within the submission (e.g., sections or figure legends), or explain why this information doesn't apply to your submission:

Group allocation is explained in Materials and Methods. Snails were selected haphazardly, and all were exposed equally to parasites. Snails were designated as infected or not depending on whether they subsequently shed parasites. Equal numbers of infected and uninfected snails were randomly chosen for pooled sequencing. Masking was not used, but it does not apply here because snails were not deliberately placed in a particular group, but rather they naturally fell into one group or the other based on infection status. Pooled sequencing, the core method for our principle discovery, is a bulk analysis that does not depend on characterizing individuals.

Additional data files ("source data")

- We encourage you to upload relevant additional data files, such as numerical data that are represented as a graph in a figure, or as a summary table
- Where provided, these should be in the most useful format, and they can be uploaded as "Source data" files linked to a main figure or table
- Include model definition files including the full list of parameters used
- Include code used for data analysis (e.g., R, MatLab)
- Avoid stating that data files are "available upon request"

Please indicate the figures or tables for which source data files have been provided:

All figures are based on raw data available at NCBI and/or other data presented in the manuscript or supplement. Scripts are available on Github.
